# Endocrine disruptor chemicals exposure and female fertility declining: from pathophysiology to epigenetic risks

**DOI:** 10.3389/fpubh.2024.1466967

**Published:** 2024-12-12

**Authors:** Sophian Tricotteaux-Zarqaoui, Marwa Lahimer, Maria Abou Diwan, Aurélie Corona, Pietra Candela, Rosalie Cabry, Véronique Bach, Hafida Khorsi-Cauet, Moncef Benkhalifa

**Affiliations:** ^1^PERITOX—Périnatalité et Risques Toxiques—UMR_I 01 UPJV/INERIS, Centre Universitaire de Recherche en Santé, CURS-UPJV, University of Picardie Jules Verne, CEDEX 1, Amiens, France; ^2^ART and Reproductive Biology Laboratory, University Hospital and School of Medicine, Amiens, France; ^3^Laboratoire de la Barrière Hémato-Encéphalique (LBHE), UR 2465, University of Artois, Lens, France

**Keywords:** endocrine disrupting chemicals, female fertility, epigenetic, endometriosis, pesticides, female reproductive disorder, infertility

## Abstract

Over the last decades, human infertility has become a major concern in public health, with severe societal and health consequences. Growing evidence shows that endocrine disruptors chemicals (EDCs) have been considered as risk factors of infertility. Their presence in our everyday life has become ubiquitous because of their universal use in food and beverage containers, personal care products, cosmetics, phytosanitary products. Exposure to these products has an impact on human reproductive health. Recent studies suggest that women are more exposed to EDCs than men due to higher chemical products use. The aim of this review is to understand the possible link between reproductive disorders and EDCs such as phthalates, bisphenol, dioxins, and pesticides. In women, the loss of endocrine balance leads to altered oocyte maturation, competency, anovulation and uterine disorders, endometriosis, premature ovarian insufficiency (POI) or embryonic defect and decreases the *in vitro* fertilization outcomes. In this review, we consider EDCs effects on the women’s reproductive system, embryogenesis, with a focus on associated reproductive pathologies.

## 1 Introduction

Founding a family, deciding the number, timing and spacing of their children are essential and inalienable human rights (1). However, the realization of these rights can be severely negated by infertility. Even though infertility is an important component of sexual and reproductive health and rights (SRHR), it remains largely neglected and underfunded worldwide (2). Infertility has devastating societal and health consequences, including severe social stigma, financial hardships, and gender-based violence, with as well as adverse physical and mental health outcomes (2, 3). Thus, addressing infertility is critical to improve the lives of individuals and couples afflicted by infertility and reach the Sustainable Development Goal 3 set by the United Nations (2, 4).

Infertility, unlike most other types of conditions, is defined by the absence of an event (i.e., not getting pregnant). The World Health Organization (WHO) defines it as “a disease of the male or female reproductive system defined by the failure to achieve a pregnancy after 12 months or more of regular unprotected sexual intercourse” (5). It affects a substantial proportion of the world’s population with approximately one in six people affected. Infertility can be primary or secondary. Primary infertility refers to the inability to achieve a first pregnancy, while secondary infertility occurs after at least one prior pregnancy has been achieved. Infertility rates are comparable for high and low-income countries (17.8 and 16.5%, respectively) and affects both men and women equally (6, 7).

Over the last 70 years, global fertility has been constantly in decline due to behavioral and societal changes (8). In fact, the number of women pursuing education and participation in the workforce has increased. Besides, the cost of raising children has become more expensive, impacting family planning decisions and leading to a late age of mothering and fathering. This delay in childbearing, which became more possible by better access to contraception, has made infertility-related disorders more difficult to treat. Literature has described the association of multiple factors with infertility such as lifestyle changing, food habits, body mass index (BMI), hormonal dysfunction, stress and late age of mothering and fathering (9, 10). Moreover, emerging evidence has shown that infertility incidence is linked to exposure to environmental factors such as tobacco, alcohol, and a wide range of endocrine disrupting chemicals (EDCs) including pesticides (Chlorpyrifos, glyphosate, dichlorodiphenyltrichloroethane and methoxychlor), phthalates, polychlorinated biphenyls (PCB), dioxins, and bisphenols (11). The association between environmental and professional EDCs exposure and male infertility has been well documented in literature and our previous published work (12). In this review, we hypothesized the link between environmental and professional EDCs exposure and women fertility declining as well as other related diseases such as endometriosis, premature ovarian insufficiency (POI) and endocrine axis dysregulation.

EDCs exposure has been reported to impact sperm and induce DNA damage and hormonal disorders in males (12). Similarly, EDCs exposure may affect female fertility at multiple levels through various mechanisms. One of the most described mechanisms is when EDCs mimic hormones such as estrogen and bind to their receptors leading to hormonal disruption (13). By interfering with the release and timing of Luteinizing Hormone (LH), EDCs can alter the ovulation process (13). Additionally, oxidative stress in ovarian tissues, that damages cells and impairs their function, is induced by several EDCs. Thus, EDCs disrupt the development of ovarian follicles and can be directly toxic to gametes, decreasing their numbers and quality (14). These molecules can also affect the epigenome causing modifications in gene expression of ovulation regulators without altering their DNA sequence (15). These mechanisms all together lead to abnormalities such as blocked Fallopian tubes, ovarian disorder, uterine disorders, failure to produce an oocyte, abnormal oocyte quality, local inflammation, and endocrine disorders in women may be linked to EDCs exposure. Failure of any or more of these conception factors can result in infertility (16). The association between exposure to EDCs and the development of potential and chronic diseases is thus a significant concern for public health, especially when fertility, a sign of health, is affected. When an individual is in subhealth, the reproductive system is more likely the first affected. This prompts us to delve in this major health problem and find a better understanding of the link between exposure to EDCs and women infertility.

The primary aim of the present review is to comprehensively summarize the current knowledge on the impact of EDCs on female fertility. This includes understanding the mechanisms by which EDCs cause ovarian aging, folliculogenesis, decrease of oocyte quality, ovulation disorders, development and receptivity of endometrium, endometriosis, fetal development abnormalities, and epigenetics modulation. Highlighting these targeted disorders by EDCs helps us shed light on taking into consideration the levels of EDCS exposure in the treatment of women’s infertility. To enhance the focus of this review, we addressed research questions about how primary mechanisms of EDCs disrupt female reproductive health in particular the impact on ovarian and follicle health, endometrium development, placenta, fetus and epigenetics. By addressing these questions, this review aims to provide a comprehensive understanding of the link between EDCs exposure and female infertility, thereby informing future research and public health strategies.

## 2 Endocrine disruptor chemicals exposure: who is concerned?

EDCs are defined by the European Chemical Agency as “chemicals that may interfere with the hormonal system and thereby produce harmful effects in both humans and wildlife” (17). EDCs are either natural or human-made chemicals that may disrupt the endocrine system, by mimicking, blocking, or interfering with the body’s hormones and may cause adverse health effects the organism or its progeny (17, 18). In fact, they alter their biosynthesis, secretion, transport, or by reducing their effects. However, the most common mechanism of action of EDCs is to imitate endogenous hormones and compete with their nuclear receptors as agonists or antagonists. EDCs have been released into the environment through human activity without prior knowledge of their impact on ecosystems or human health. Consequently, different national or supranational agencies established lists such as REACH of known or suspected EDCs, chemicals exhibiting effects on the endocrine system (19). Chemicals considered as potential EDCs will be subject to further evaluation and regulation to reduce their use and ultimately replace them with safer alternatives. Over the years, public concerns grew toward EDCs and recent studies have suggested that EDCs are associated with cancer (20), cardiovascular risk, behavioral disorders, autoimmune abnormalities (21), and reproductive disorders (18, 22).

**Figure 1:**
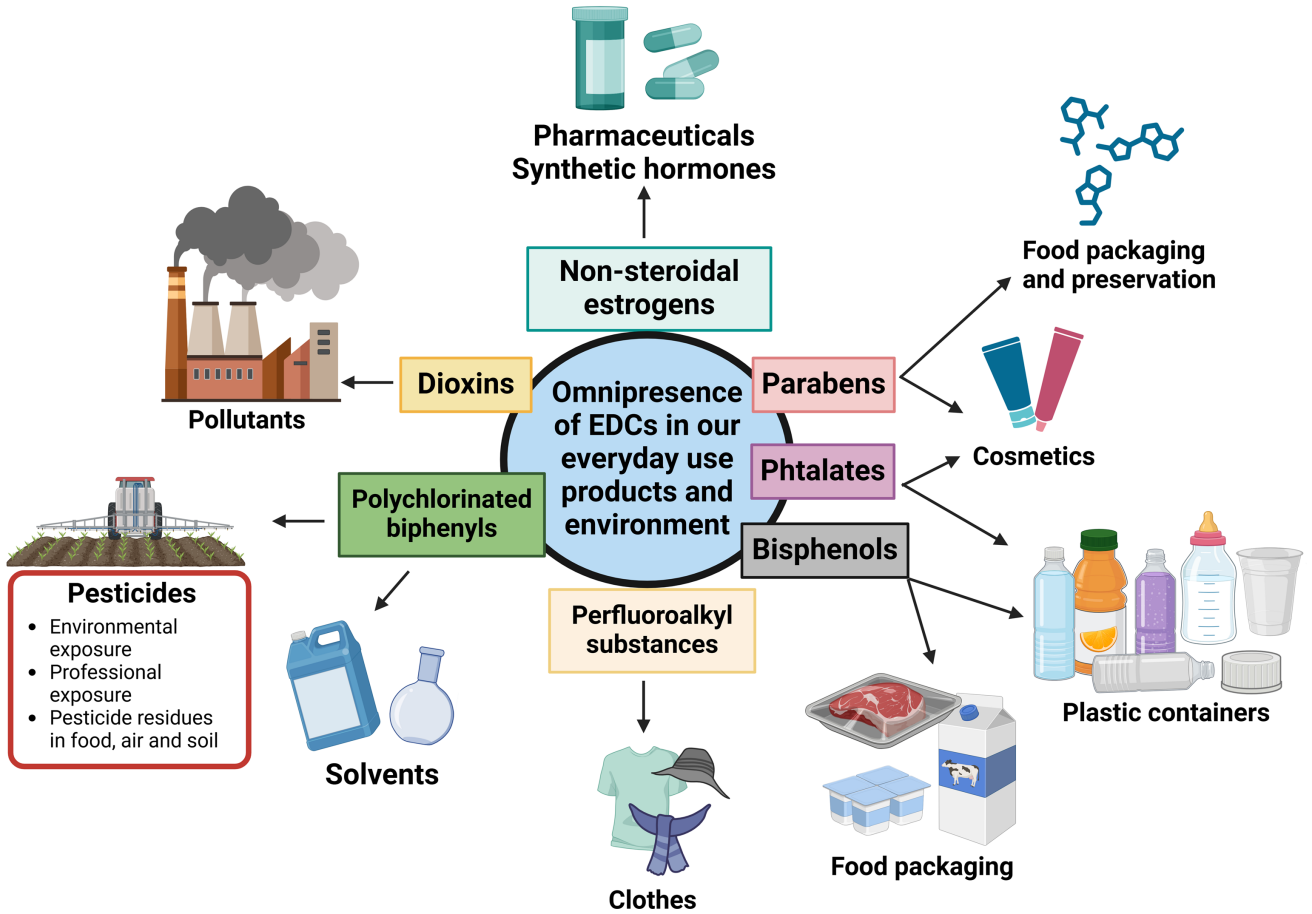
Various groups of endocrine-disrupting chemicals present in daily life. Adapted from Yan et al. (25).

Humans are chronically exposed to a wide range of EDCs. Their exposure is related to their lifestyle and profession. In high producers and pesticide consumer countries, mostly in United States and Europe, chemical industries workers and especially farmers are highly exposed to pesticides that have been identified as EDCs. These cases are mostly detected in regions with high agriculture activities. As an example, in the Haut De France region in northern France, known for its extensive use of pesticides to maintain high agricultural output, studies of the impact of EDCs showed that altered sperm parameters (23) and oocyte dysmorphism (24) are associated with exposure to pesticides in risk zones. EDCs exposure does not only concern professionals. By contaminating food, soil, and the air, these substances reach the general population (by ingestion, inhalation, and skin contact) and expand to an environmental exposure. As food itself contains ECDs residues, many other everyday items, such as plastic bottles, food containers, the liners of metal food cans, detergents, flame retardants, gadgets, cosmetics, contains chemicals such as PCB, bisphenol A (BPA), phthalates and their metabolites (dibutyl phthalates (DBP) and di-(2-ethylhexyl) phthalate (DEHP)), and alkyl phenols, that are also classified as EDCs (25) (Figure 1).

However, exposure degree differs between individuals due to different lifestyle, food habits, and habitat location. In addition, the dose, the route of exposure, and the individual’s susceptibility are also key factors to determine the impact of EDCs on reproductive health. Moreover, the timing of exposure in an important parameter to evaluate the effect of EDCs. As the DOHaD concept explains, when the exposure occurs in the first 1,000 days of life, the risk of developing pathologies in adulthood is higher than exposure occurring during adult age (26, 27). A prime example is diethylstilbesterol (DES) a potent synthetic estrogen, prescribed to pregnant women to prevent miscarriage. *In utero* exposure to DES has been associated with reproductive tract abnormalities and the development of vaginal clear-cell adenocarcinoma in young female offspring (28) and breast cancer (29). The effects of these chemicals on the endocrine system had devastating consequences on individuals. However, almost all major classes of EDCs can target the estrogen pathways as many EDCs display estrogenic activity (30, 31), and can affect both genetic (32) and epigenetic levels. Thus, the hypothalamo-pituitary-gonadal (HPG) axis in women is a prime target of EDCs.

The HPG axis is a hormone-regulating structure crucial for reproduction. There are noticeable distinctions in how the HPG operates in males and females. Unlike its male counterpart, the female reproductive system shows regular cyclic changes that can be regarded as preparation for fertilization and pregnancy. Two cycles occur at the same time: the ovarian cycle and the uterine cycle. The ovarian cycle features the series of changes happening within the ovary with follicles recruitment, the oocyte maturation and release, and the *corpus luteum* development before regressing. The uterine cycle features endometrial thickening in preparation for embryo implantation, as well as the shedding of the uterine lining following unsuccessful implantation. They are both regrouped into a single cycle describing all these physiological changes that occur, the menstrual cycle (33). The menstrual cycle is tightly controlled by four structures that communicate with each other’s and orchestrate this cycle: the hypothalamus, the pituitary, the ovaries, and the uterus. The key hormones of this system are the ovarian hormones estradiol (E2) and progesterone (P4), the gonadotropin-releasing hormone-I (GnRH) secreted within the hypothalamus, the gonadotropins luteinizing hormone (LH) and follicle-stimulating hormone (FSH) secreted in the pituitary. P4 and E2 will modulate the activity of the neuronal network in the hypothalamus controlling the release of GnRH that will be secreted into the pituitary portal circulation in an intermittent manner. This intermittent release of GnRH will generate very distinct pulses of LH and FSH, allowing the appropriate development of the recruited follicles during the follicular phase (34). The frequency and amplitude of GnRH pulses are determined by multiple neuroendocrine modulators including kisspeptin (KISS), leptin, ovarian steroids, and peptides providing a link between the environment and reproductive status. Recently, emerging studies have shed light on a key reproductive neuroendocrine modulator KISS and EDCs exposure. KISS stimulates endogenous GnRH secretions and is involved in both E2 positive and negative feedback (35). A French study using animal experimentation assessed the impact of maternal exposure to a pesticide chlorpyrifos (CPF) and a high-fat diet (HFD) on the reproductive function of rat offspring. The study outcome revealed that pesticide exposure disrupts the normal expression of KISS and GnRH, in the testis, potentially leading to reproductive health issues. Additionally, histological analysis showed a significant increase in atretic follicles and abnormal testis structures with germ cell desquamation in the CPF-exposed group. This suggests the role of kisspeptin in reproductive disorders (36). These signals provide a link between the environment and reproductive status. The HPG plays a crucial role in the regulation of the reproductive function in women. Any alteration of the functionality or imbalance on this beautifully orchestrated system will have adverse or deleterious effects on women’s reproductive health and thus may lead to infertility.

## 3 Effect of EDC on ovarian aging and decrease of oocyte quality

### 3.1 Ovarian aging

It is generally accepted that women are born with a finite follicle pool, called the ovarian reserve, that will go through a constant decline as they age and will ultimately lead them to the final step of their fertile life, the menopause. Ovarian aging is a natural and physiological process, which encompasses a progressive and gradual decline in both quantity and quality of oocytes within primordial follicles of the ovarian cortex until exhaustion of ovarian function (37, 38). This gradual decline will impair the reproductive ability of women as they age by demonstrating diminished fertility early in their life (about 35 years old) due to decay in oocyte quality and will cause endocrine dysfunction and raises their risks of cardiovascular diseases once they reach the final stage of ovarian aging (39, 40).

POI is a serious medical condition characterized by very low blood levels of anti-Müllerian hormone, where the ovarian reserve is depleted before the age of 40, leading to a premature loss of ovarian function and early menopause. This acceleration of follicular atresia often results in subfertility or infertility associated with menstrual irregularities and high pregnancy failure (41, 42). This condition affects 1% of women worldwide and alters severely their quality of life. Even though genetic abnormalities have been associated with POI, its etiology remains undetermined in more than 75% of cases. However, the environmental factor seems to be the main cause beyond the genetic reason (43).

Over the last few decades, evidence has confirmed that exposure to EDCs was a major environmental risk factor for ovarian function, ovarian aging, and POI. Indeed, several studies have linked exposure to persistent EDCs, such as dioxins, organochlorine pesticides (e.g., DDT) (44), PCBs (45), perfluoroalkyl and polyfluoroalkyl substances (PFASs) (46), and non-persistent EDCs, phthalates, phytoestrogens, BPA, and parabens to ovarian aging. A French study associated pesticides exposure with an endocrine-disrupting action in agricultural region with syndromes linked with fertility decline, poor IVF outcomes, such as POI, polycystic ovarian syndrome, and endometriosis (47).

The case of phthalates is remarkable concerning ovarian aging. Phthalates are groups of synthetic chemicals widespread in everyday products such as plastic containers, drug coating, building, and personal care products that lead to ubiquitous human exposure via oral ingestion, inhalation, and direct contact. Furthermore, women are often exposed to higher levels of phthalates than men through their extensive use of personal care and cosmetic products (48). Phthalates are described in many studies as being endocrine disruptors altering ovarian function. One way that phthalates can exert ovarian toxicity rests on one of the most commonly found active metabolites of phthalates, the mono-(2-ethylhexyl) phthalate (MEHP). MEHP induces an oxidative stress [imbalance of pro- and antioxidants in a system mostly induced by raises the physiological level of reactive oxygen species (ROS)] within antral follicles, increasing the expression of proapoptotic factors such as *Bax*, which inhibit the follicle growth. It also reduces the expression of cell-cycle regulators (*Ccnd2*, *Ccne1* and *Cdk4*), the antiapoptotic factor (*Bcl-2*) and thus, reducing estradiol (E_2_) production by inducing atresia (14).

**Figure 2:**
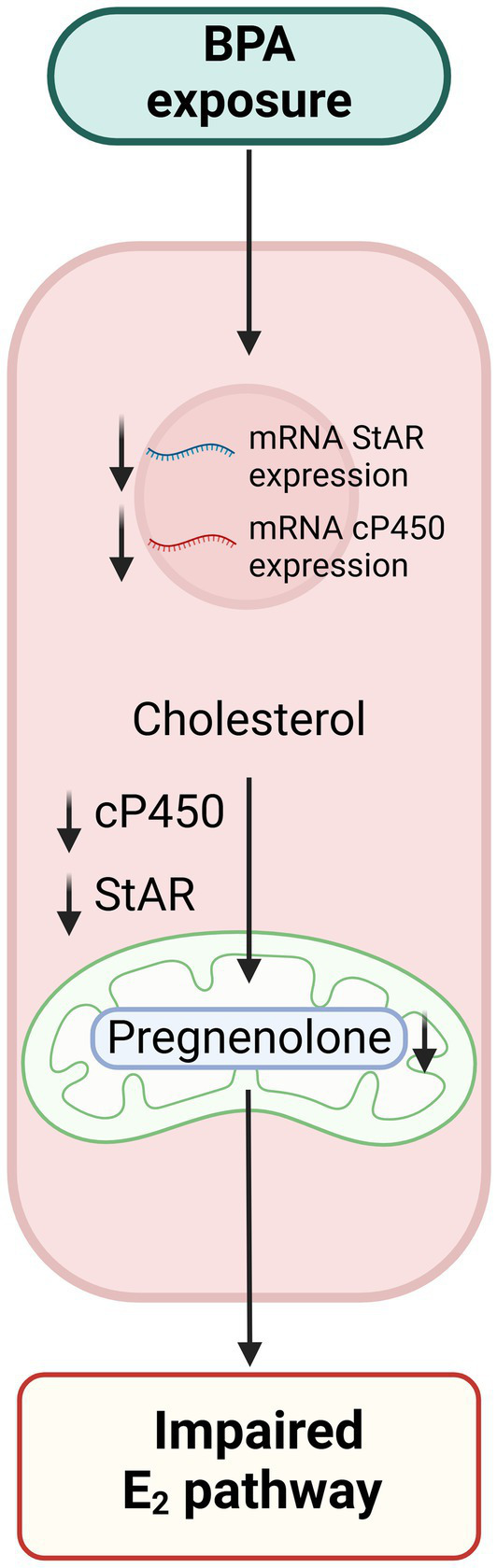
Suggested mechanisms of Bisphenol A impairing decidualization. Exposure to BPA leads to downregulation of steroidogenic acute regulatory protein (*StAR*) and cP450 expression in theca and granulosa cells. This downregulation hinders the transport of cholesterol into the mitochondria for conversion into pregnenolone, thereby disrupting the E2 biosynthetic pathway and impairing the normal function of the HPG axis.

BPA, another domestic EDC mostly find in food and beverage packaging, is a known estrogen mimicker binding on the estrogen receptor *α* (49). Studies in mouse models pointed out that exposure to BPA is associated with a decrease in ovarian reserve regardless of the exposure window whether it is prenatal, neonatal or adult age (50). Indeed, by inhibiting steroidogenesis, BPA decreases crucial hormone production in antral follicles, leading to an inhibition of follicle growth and induces atresia. In the granulosa and theca cells, BPA down-regulates the steroidogenic acute regulatory protein (*StAR*) and the *cP450* sidechain cleavage mRNA expression preventing cholesterol transport into the mitochondria and impairs the E_2_ biosynthetic pathway altering the HPG normal function (Figure 2) (51). Normally EGs, such as E_2_, stimulate follicle growth and protect the follicle from atresia but it is no longer the case in presence of BPA (52).

### 3.2 Impact on folliculogenesis and oocyte quality

In addition to affecting ovarian aging mechanisms, EDCs can exert ovarian toxicity through targeting granulosa and theca cells, which are crucial for oocyte development. Some broad-spectrum pesticides are also known as EDCs, such as the renowned DDT, it’s subsequent dehydrochlorination the DDE, and methoxychlor (MXC) will impact the folliculogenesis. DTT and DDE, have been well studied from the 90s since Guillette et al. report about altered sex steroid hormone concentrations in alligators (45). DDT and DDE both shown alter progesterone biosynthesis in a dose-dependent manner and impact E_2_ production by decreasing cAMP synthesis, thus interfering with gonadotropin receptor signaling pathways in granulosa and theca cells. Many studies have demonstrated that their replacement, MXC and its metabolite, have also adverse effects on progesterone synthesis, E_2_’s FSH-stimulated biosynthesis and increase E_2_ catabolism in granulosa and theca cells leading the either follicular atresia or to meiotic aberrations (aneuploidy) in oocytes impairing its quality (47).

Another way for EDC to impact oocyte quality and development is via DNA damage through oxidative stress. Oocyte quality is defined as the competence of an oocyte to develop a genetically undamaged embryo. Liu et al. (53) suggested that MXC exposure induces oxidative stress and affects mouse oocyte meiotic maturation via the accumulation of superoxide radicals and other ROS by decreasing the enzymatic activity and expression of antioxidants SOD1, GPX and catalase.

The mitochondria, the main source of ROS, is also a target for EDCs. Growing evidence shows that mitochondrial imbalances are associated with impaired oogenesis, poor oocyte quality and ovarian aging. The loss of energy balance and damage to the mitochondria induce mitochondrial stress increasing ROS level that leads to apoptosis and thus, depleting the ovarian reserve (54, 55).

As a result, this evidence emphasizes that the dysfunction of the HPG axis by altering the ovarian function is a contributing factor to ovarian aging and oocyte quality loss in humans.

## 4 Effect of EDC on ovulation

EDC can influence the fertilization process in women by different means. If the oocyte cannot mature, due to an early ovarian aging, impaired folliculogenesis or cannot be expelled due to anovulation, the fertilization cannot occur. Moreover, if the released oocyte quality is impaired due to altered maturation, the fertilization can occur, but oocytes with cytoplasmic anomalies will lead to significantly lower pregnancy rates (56).

The WHO has developed a classification standard in 1973 for anovulation diseases with 3 categories.

Group I (Hypothalamus–Pituitary Failure). This situation may be caused by low LH and FSH concentration and with reduced E_2_ levels. In this case the development of follicles in the ovary is blocked, which in turn leads to anovulation and amenorrhea.

Group II (Hypothalamic–Pituitary Dysfunction). This is the most common type of anovulation, accounting for about 90% of all anovulatory patients. In this type of anovulation, women’s serum gonadotropin and E_2_ levels are normal, but LH/FSH are often abnormally elevated. The occurrence and growth of follicles can be observed, but the follicles cannot mature and cannot spontaneously ovulate.

Group III (Ovarian Failure). This condition is mainly due to severe lack of primordial follicles with increased serum FSH and LH, and decreased E_2_. The clinical manifestations are characterized by a poor response to induced ovulation, and the ovarian function has deteriorated. The patients suffer from amenorrhea, infertility, and low estrogen-induced perimenopausal symptoms (57, 58).

While anovulatory disorders may be caused by drug uses, genetic factors, or chronic diseases, they are one of the main manifestations of EDC on the women’s reproductive health. Ovulation is a biological process that is so finely tuned, any imbalance in the HPG axis and the endocrine system surrounding it may lead to ovulatory blockade. Thus, impaired folliculogenesis induced by BPA, a high aneuploidy rate induced by MXC, or the acceleration of follicular atresia as described above, will lead to anovulation due to the failure of the oocyte to reach maturity.

Atrazine (ATR) is a widely used herbicide used for agricultural purposes in the United States, China, and Africa and a common environmental contaminant known with EDC effect and reproductive toxicity (59). Its anovulatory effects have been demonstrated throughout different animal studies. In female rats, ATR reduced the number of oocytes released, the effect of LH surge by suppressing FSH-induced LH receptor expression and as well as the length of estrus cycle. In FSH-stimulated rat granulosa cells, ATR reduced serum E_2_ serum levels and reduce LH receptor mRNA levels, resulting in ovulation blockade (60). Studies in human granulosa cells have also demonstrated that ATR reduces the levels of E_2_ and (progesterone) P_4_ and prevent LH-dependent expression of ovulation genes (61).

Hypothalamic hormones are also important factors responsible for a normal ovulation. Thus, any disturbance on the GnRH production or pulse frequency caused by EDC that incurs a slower or faster frequency than the physiological cycle of 60–90 min, altering follicles development in the ovary which in turn leads to anovulation (62).

## 5 Effect of EDC on uterine development, endometrial receptivity, and implantation

**Figure 3:**
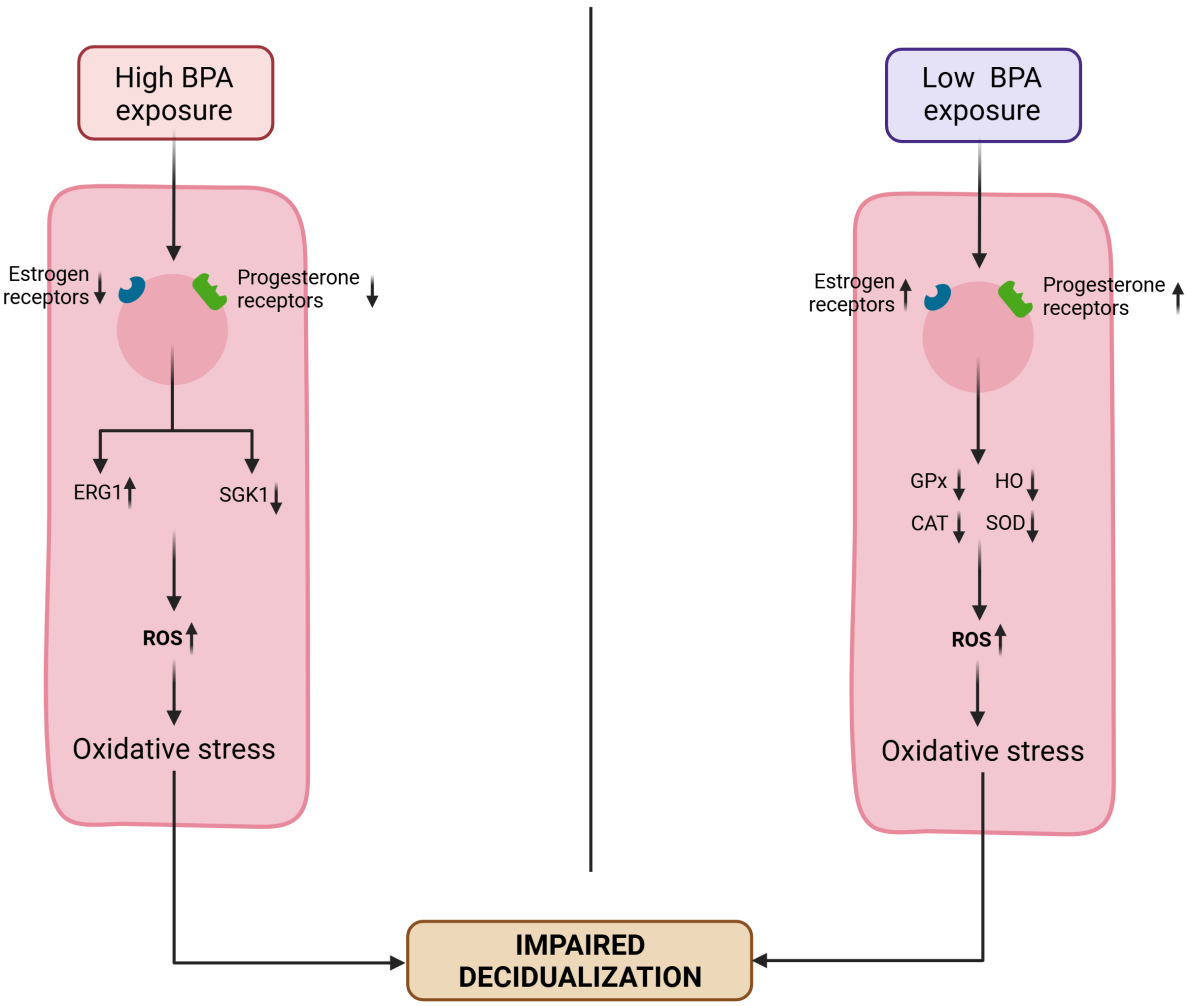
Suggested mechanisms of Bisphenol A impairing decidualization. Exposure to low doses of BPA leads to an upregulation of estrogen (ER) and progesterone (PR) receptors. This reduces the levels and activities of antioxidant enzymes (superoxide dismutase [SOD], glutathione peroxidase [GPx], heme oxygenase [HO], and catalase [CAT]), and thus increasing ROS level leading to an impaired decidualization. Exposure to high level of BPA downregulates the ER and PR, increase the expression of early growth response-1 (EGR1) and reduce the expression of glucocorticoid-induced kinase-1 (SGK1), resulting in elevated ROS levels, which in turn impair decidualization. Adapted from Nelson et al. (64).

The endometrium is a dynamic layer that undergoes proliferation, secretion and shedding phases during the menstrual cycle and involves a balanced yet complex interaction between E_2_ and P_4_. EDCs by altering the endocrine equilibrium within the HPG axis can affect this crucial uterine layer and evidence has confirmed that exposure to EDCs was a major environmental risk factor for uterine disorders. Studies have shown that, the process of decidualization is sensitive to EDCs and may pose a hazard to embryo attachment and implantation (63). BPA induces impairment of decidualization through the dysregulation of EG and P_4_ receptors in endometrial cells. At low BPA exposure, decidualization is impaired by oxidative damages due to an increased level of ROS. By upregulating EG receptors and P_4_ receptors, antioxidant enzymes such as SOD, GPx, HO, and catalase have reduced activities, and nitric oxides see their production increased. At high levels of BPA exposure, EG and P_4_ receptors are down-regulated and impairs decidualization by increasing ERG1 expression and decreasing SGK1 serum level (64) (Figure 3). The disruption of their function could lead to implantation and/or placentation failure leading to pregnancy complications, loss of pregnancy, or infertility (65, 66).

Other EDCs such as endosulfan reduces the expression of the primary endometrial markers of receptivity (such as MUC1, HOXA10, Inn and E-cadherin) and affect the normal endometrial receptivity impairing the adhesion and the implantation process of the blastocyst (67). Emerging evidence shows that EDCs can also affect the receptivity of the endometrium by disturbing the immune system that plays a critical role in the events of implantation, early placentation and alter the endocrine signaling required for maternal immune tolerance toward implantation and thus altering the timing of the implantation window (68). A successful implantation requires synchronized actions of both the blastocyst and the endometrium to attach and initiate the implantation.

## 6 EDC and endometriosis risks

Endometriosis is a chronic estrogen-dependent inflammatory systemic gynecological disease, where alterations involving both the endocrine and immune systems have been observed. Clinical symptoms are often life-impairing pain and infertility and affects approximately 10% of women in reproductive age worldwide. This common yet complex disease is difficult to diagnose as most of the time the disease is found during a diagnostic work-up for infertility. Furthermore, laparoscopy or surgery is required in order to make the definitive diagnosis. Even though endometriosis is currently actively researched, its etiology is still today poorly understood. A combination of hormonal, metabolic, genetic, epigenetic and environmental factors are currently being investigated. However, growing evidence suggests that EDCs exposure may be a co-causal factor that could play a role in disease etiology. By their ability to alter steroidogenesis, EDCs could affect endometriosis. In fact, suspicions about ECDs involvement first arose from a study conducted by Rier and his coworkers in 1993 (69), where they observed that the severity of endometriosis in Rhesus monkey was directly correlated with TCDD exposure. TCDD is known to disturb EG and P_4_ pathways, and as an estrogen-dependent disease, E_2_ production and P_4_ resistance play a crucial role in the disease progression and/or maintenance. TCDD also unbalance the pro/anti-inflammatory balance and activates an inflammation-like pattern using the AhR pathways that could be involved in the development and progression of endometriosis by increasing vascularization, cell proliferation, and VEGF levels (47, 70). Increased local production of E_2_ can also promote endometriosis in combination with TCDD inhibition on P_4_. Furthermore, women afflicted by endometriosis demonstrate decreased responses to P_4_. Short term exposure to TCDD reduced endometriotic lesions due to its estrogen antagonist proprieties (71), highlighting the effect of long chronical exposure to develop the disease.

## 7 Effect of EDC in fetal development

### 7.1 Impact on the placenta

The placenta is an ephemeral yet vital organ that performs various endocrine, immunological, and physiological processes throughout pregnancy. It develops from the trophectoderm layer of a blastocyst through placentation (72). Its role is essential for the normal development and growth of the fetus as it produces hCG to maintain pregnancy, is the primary site of the transport of nutrients, gases, and waste materials between the fetus and the mother, and acts as a barrier protecting the fetus during pregnancy. Its failure has devastating consequences. Placenta anomalies lead to pre-eclampsia, abnormal fetal development, miscarriage, and placental disruption. The placenta is vulnerable to endocrine disruption due to a large presence of hormone receptors and a lack of enzymatic machinery to guard against EDCs (25). Evidence suggests that phthalates such as DEPH, alter placental endocrine function, and inhibits placental cell proliferation by decreasing the expression of trophoblast differentiation markers (PPARγ, AhR, hCG) (73, 74). Furthermore, phthalate exposure disrupts the vascularization of the placenta, induces apoptosis of placenta cells reducing placental size and shape (75). BPA exposure alters nutrient transport, particularly glucose transporter by affecting GLUT1 expression level (76–78) and exhibit trophoblast cell invasion, and abnormal placental vessel remodeling in mice (79). Other EDCs such as organochlorine pesticides can impair placenta ability to produce and release hormones and enzymes, transport nutrients, or produce waste. They contribute to preterm birth by disrupting the balance between P_4_ and E_2_ during pregnancy (25).

### 7.2 Impact on fetuses/embryo

Impact of EDCs can go beyond the placenta and reach the fetus. Immature fetuses are vulnerable to EDCs exposure because different EDCs such as BPA, dioxins, and organochlorine pesticides can breach the placental barrier using passive diffusion and can be transferred from the mother to the fetus. As EDCs perturb the endocrine system, EDCs exposure in humans is highly harmful during these critical life stages as early development requires dose and accurate timing of hormone action to endorse proper tissues and organs growth and development. However, just like the placenta, fetuses typically lack the enzymatic machinery to guard against EDCs effects as elimination processes are not fully developed (80, 81). During early embryonic development, EDCs such as dioxins (TCDD) can target cell cleavage, differentiation, or organogenesis (82). Other EDCs, such as DDT and BPA were linked to impaired fetal growth, and metabolic dysfunction which can possibly lead to miscarriage (25, 83).

The impact of EDCs extends beyond lowering the rate of a successful pregnancy and increasing the risk of miscarriage in women; they also impair the future reproductive health of the fetus. EDCs exposure during this critical developmental window can have lasting health effects on the fetus, extending into adulthood. Thus, EDCs such as BPA exhibits pseudo-estrogenic and anti-androgenic effects, which may disrupt the developmental program of the male reproductive system. In mice, BPA exposure during pregnancy, resulted in decreased testosterone secretion, sperm motility and sperm count in male offspring (12, 84, 85). Studies on female offspring have shown that *in-utero* exposure to BPA impaired early oogenesis and follicle formation in the fetal ovary (86, 87).

## 8 Impact of EDC on epigenetic risks

Embryo development stages are part of the prenatal period that is a sensitive window for potential environmental factors negative effects. During this period, vulnerability to pollutants is higher than in adults. Exposure to toxic molecules during this sensitive window is a risk factor to chronic disease in adulthood. In fact, the embryo is deprived of any detoxification or excretion mechanism (88). As a result, the reach of EDCs to the embryo by transplacental transfer can lead to genome alteration during embryonic germ cell precursors reprogramming. As DNA has evolved to build resistance against external threats to preserve the stability of the genome, EDCs mainly affect the epigenome (89). Thus, genetics and environmental pollutants are indissociable risk factors of infertility which is clearly a complex multifactorial disorder (90). Epigenome alterations by EDCs include DNA methylation patterns (at the fifth carbon of the cytosine base, 5-methycytosine), histone modification and miRNA regulation, as previously detailed in our published work regarding epigenetic modifications and male infertility (12). EDCs can deregulate epigenetic patterns by altering methylation enzymes (DNMTs) and DNA demethylation enzymes (e.g., ten-eleven translocation TET proteins) (15). Resulting transcriptional changes may explain all the reproductive disorders mentioned above and the negative influence on human fertility. In fact, the establishment of the DNA methylome is crucial for the beginning of the ovarian cycle during embryo development stages since DNA methylation regulates gene expression to control cell differentiation (91). Research has shown that exposure to the synthetic estrogen DES can cause epigenetic modifications that affect not only the exposed individuals but also their offspring. Reproductive tract abnormalities in women have been linked to alterations in DNA methylation patterns that control several estrogen-responsive genes in addition to histone modifications (89), after exposure to DES. Furthermore, DES prenatal exposure induced a decrease in *Tet1* mRNA expression in mouse uterus while BPA exposure leaded to its down regulation in zebrafish gonads. Interestingly, studies suggest that BPA effect can be sex-specific: prenatal exposure led to DNA hypermethylation in male offspring but DNA hypomethylation in female (92). Altered DNA methylation patterns by BPA lead to impaired folliculogenesis and reduced oocyte quality. These changes can persist into adulthood, leading to long-term fertility issues (92). In addition to their impact on methylation and demethylation enzymes, DES and BPA can also affect cofactor (methyl donor) of these enzymes (15). In parallel, DES and BPA have been shown to change histone-modifying enzymes (93). Epigenetic changes linked to BPA exposure have been associated with early puberty in girls (94). Another consequence of DNA methylation changes is inhibition of mammalian oocytes maturation. Moreover, epigenetic modifications by dioxins and phthalates decrease antral follicle counts and lead to aberrant folliculogenesis (89). Exposure of mice to phthalates doses close to human exposure caused multigenerational effects on ovaries (94).

Many biological processes depend on differentially methylated regions known as imprinted control regions (ICRs) that are established during gametogenesis (89). Modification of methylation pattern of these regulatory sequences can prevent silencing of potentially dangerous transposons and proper chromosome segregation. Exposure to BPA has been linked to 1,251 human transposon regions that were differentially methylated (95). Further differential DNA methylation caused by EDCs (PCBs DEHP, methyl mercury and pesticides) has been detected in many locus/genes (96). Notably, phthalates exposure has been linked to differentially methylated regions of genes involved in early growth and metabolic processes. It has been demonstrated that phthalate BBP (butyl benzyl phthalate) can modulate the methylation of the estrogen receptor (ESR1) promoter site, altering the transcriptional expression (97). Moreover, they can modulate microRNAs, which means that these substances are able to interfere with more than one epigenetic process and have transgenerational effects at F1 and F3 generations (98, 99). Exposure to the phthalate DEHP during the prenatal period induced dysregulation in hormone levels and folliculogenesis and led to a multigenerational increase in ovarian cysts. Exposure to DEHP in ancestors accelerates the onset of puberty and reproductive aging in the F3 generation of female mice (100). Similarly, methoxychlor and DDT pesticide exposure during gestation was linked to incidence of ovary diseases in the F1 and F3 (101, 102). Even though these pesticides have been banned, differentially methylated regions they induced expanded their effect to the next generations: sperm epimutations in parent resulted in obesity and ovarian dysfunctions in female offspring (103). These epigenetic modulations by EDCs suggest serious long-term effects on human reproductive health and fertility decline.

The clinical and research communities have lately given great interest in the monitoring and reversing of epigenetic changes induced by EDCs. DNA methylation can be monitored using bisulfite sequencing and methylation-specific PCR while histone modifications can be detected using chromatin immunoprecipitation (ChIP) assays. In addition, RNA sequencing and microarray analysis can measure the expression of non-coding RNA affected by EDCs (104). Notably, pharmacological interventions, including the use of DNA methyltransferase inhibitors and histone deacetylase inhibitors, have demonstrated potential in reversing epigenetic modifications (105). Additionally, lifestyle and dietary changes, such as the intake of specific nutrients and antioxidants, have been suggested by some studies to help mitigate the epigenetic effects of EDCs (106, 107).

Today, EDCs are ubiquitous and exposure to these substances is now nearly impossible to avoid. Highly used and produced for the effect they were intended for, these environmental pollutants are now everywhere, and the general population is exposed through food and beverages, air, water and ground. Some populations are more exposed to these pollutants than others. Indeed, farmers, hairdressers, cashiers, to quote a few, are not only exposed in their private life but they are also exposed through their professional life. With the decline of global fertility, the impact of exposure to various EDCs on reproduction is now a public health concern. Studies showed that they are associated with male and female fertility decline, low ongoing pregnancy rates, and elevated risk of miscarriage. However, a lot of crosstalk toward EDCs still happens. It’s not rare to encounter situations where known EDCs are still in use because the advantages, they offer exceed the health risks associated. Many EDCs can interact with the female reproductive system in early development, in puberty but also during their adulthood and may lead to various reproductive disorders due to endocrine disruption. EDCs effect has more deleterious effect on women than men, as severe endocrine disorders may lead to an early end of their reproductive life or can severely impair their ability to carry out a pregnancy. Their reproductive system is especially vulnerable to compounds that mimic estrogen or alter the estrogen pathway. Scientific research on the impact of EDCs on the reproductive system and fertility has yielded complex and sometimes conflicting evidence due to the very diverse mechanisms of action of EDCs and the challenges of studying real-life mixtures. Furthermore, limitations in current studies, including variability in exposure levels and the complexity of human reproductive systems, make it challenging to fully understand EDCs’ impact on human fertility. However, the negative impact of EDCs on female fertility is now a global health concern that must be addressed to improve the fertility of affected individuals and couples. Even though today ART techniques are quite developed, they are not miracle techniques able to resolve any female infertility caused by EDCs. Thus, from a clinical perspective, evaluating EDCs exposure, particularly professional exposure, and screening for these chemicals could assist practitioners in more effectively managing couples seeking infertility treatment. On the other hand, future research on EDCs should aim to collect multiple exposure windows, consider the presence of EDC mixtures in order to fully comprehend the impact of EDCs on female fertility and take in account the professional exposure of these couple coming for infertility treatment.

## 9 Conclusion

**Figure 4:**
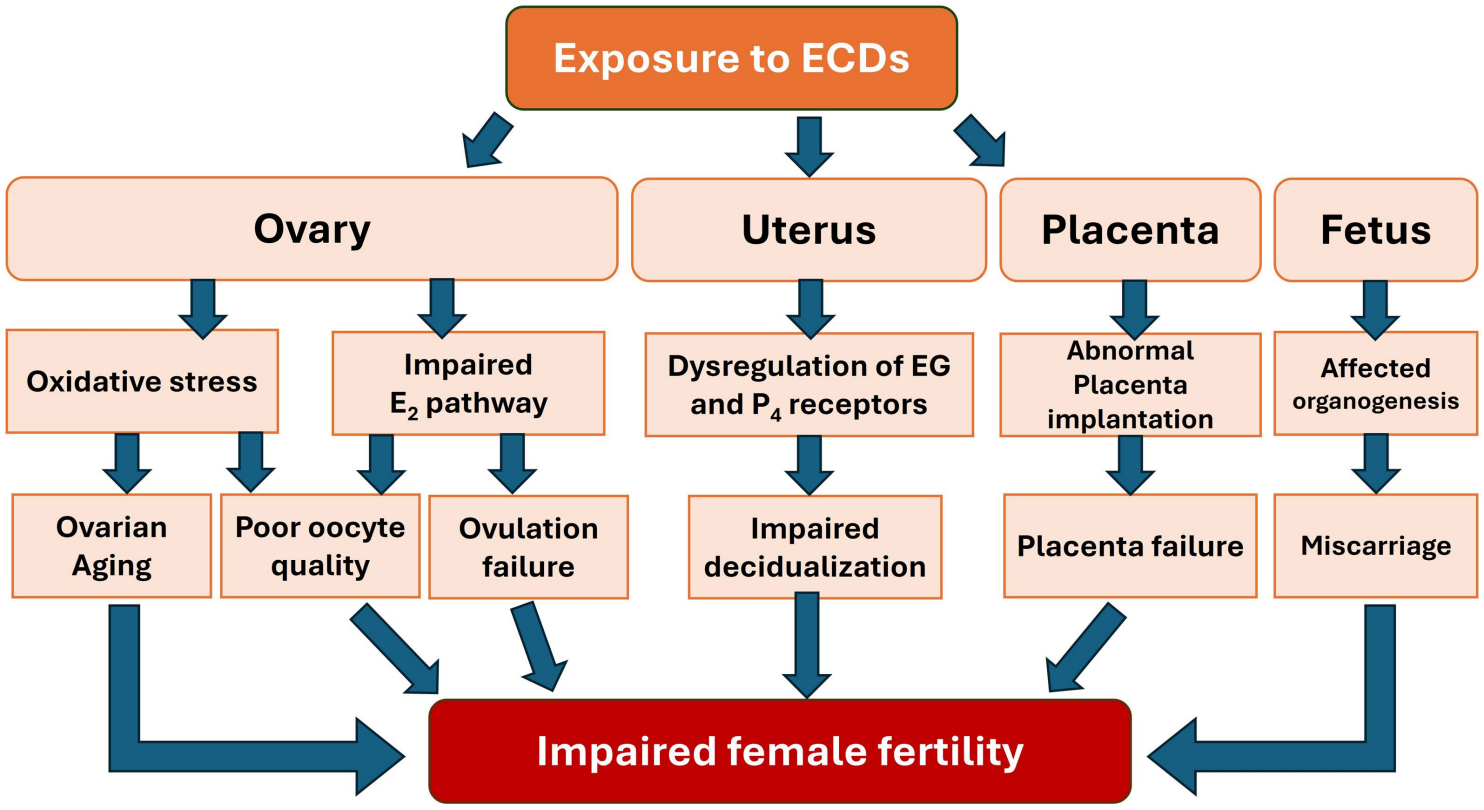
Impact of EDCs exposure on the reproductive system and fetal development associated with impaired female fertility.

In summary, the impact of EDCs on female reproductive health is multifaceted, affecting various processes from ovarian aging and folliculogenesis to endometrial receptivity, ovulation, and implantation. Our review highlights how EDCs disrupt these key reproductive events through mechanisms such as interference with hormonal signaling pathways, the hypothalamic–pituitary-gonadal (HPG) axis and epigenetic modifications. The detailed examination of these mechanisms underscores the complexity of EDCs’ effects, linking them to clinical outcomes like reduced IVF success rates. Additionally, our discussion on fetal development and epigenetic risks underscores the critical importance of these areas in understanding the full scope of EDCs’ impact. By expanding on these topics, we aimed to provide a more comprehensive view of how EDCs influence not only immediate reproductive processes but also long-term generational health. This integrated discussion underscores the importance of considering the cumulative and interconnected effects of EDCs on female fertility, offering a holistic perspective that bridges the detailed sections of our review (Figure 4).
